# Robust ultrahigh-Q resonances in tetramer metasurfaces through centroid symmetry protection and area conservation

**DOI:** 10.1038/s41377-025-02164-7

**Published:** 2026-01-23

**Authors:** Chaobiao Zhou, Rong Jin, Haoxuan He, Jing Huang, Guanhai Li, Lujun Huang

**Affiliations:** 1 School of Physics and Mechatronic Engineering, Guizhou Minzu University, Guiyang, 550025 China; 2 State Key Laboratory of Infrared Physics, Shanghai Institute of Technical Physics, Chinese Academy of Sciences, Shanghai, 200083 China; 3 Hangzhou Institute for Advanced Study, University of Chinese Academy of Sciences, Hangzhou, 310024 China; 4 Shanghai Research Center for Quantum Sciences, Shanghai, 201315 China; 5 State Key Laboratory of Precision Spectroscopy, School of Physics, East China Normal University, Shanghai, 200241 China

**Keywords:** Nanophotonics and plasmonics, Metamaterials

## Abstract

Ultrahigh-Q optical resonances are the cornerstone of next-generation nanophotonic technologies, but their simultaneous realization of robustness and on-chip practicality remains a significant challenge. In this work, we present tetramer composite metasurfaces capable of supporting two distinct classes of ultrahigh-Q resonances: centroid symmetry-protected bound states in the continuum (SP-BICs) and area-conserved guided-mode resonances (GMRs). By employing a four-hole supercell design, we demonstrate that centering each hole within its subcell preserves C_4v_ symmetry, thereby enabling SP-BICs. Controlled lateral displacement transforms them into quasi-BICs with Q > 10⁶. Independently, enforcing diagonal-hole area conservation within the super unit cell generates degenerate GMRs with Q > 10⁸, which exhibit remarkable stability across a broad wave vector range. Breaking this area conservation splits the GMRs into paired ultrahigh-Q resonances, while adjusting the center-to-center distance of air holes lifts their degeneracy. Experimentally, we validate both resonance types using silicon photonic crystal slabs, achieving measured Q-factors exceeding 10,000, with a maximum value of 43,700. Such ultrahigh-Q composite-metasurfaces provide a versatile platform of enhancing light-matter interactions.

## Abstract (web summary)

We report two distinct classes of ultrahigh-Q resonances-centroid symmetry-protected bound states in the continuum, and area-conserved guided-mode resonances in tetramer metasurfaces.

## Graphical abstract

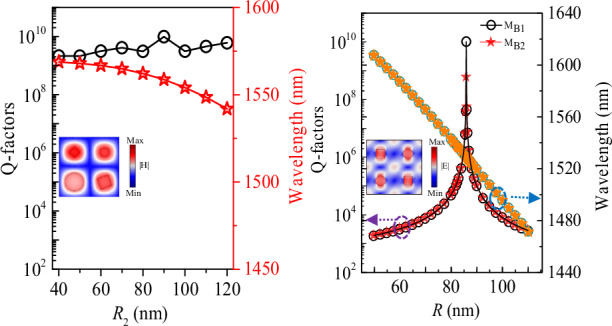

## Introduction

In recent years, there has been an increasing interest in dielectric metasurfaces because they offer a novel platform for the development of high-performance nanophotonic devices^1^. Typically, all-dielectric metasurfaces support multipolar Mie resonances and have much lower intrinsic loss than noble metals^2^. Also, they are compatible with CMOS processes. Recent studies show that low-loss dielectric metasurfaces also support various optical radiation-free modes, such as bound states in the continuum (BICs) and guided modes (GMs). These modes are usually characterized with infinite quality (Q) factors, thus providing an easy way of realizing ultrahigh-Q resonances and promising widespread applications in the field of nanophotonics^3,4^. Notable applications include ultralow-threshold nanocavity lasers^5^, enhanced nonlinear harmonic generations^6^, quantum sources^7^, strong coupling phenomena^8^, biosensor^9^, thermal emission^10^, and electric-optic modulators^11,12^.

Essentially speaking, BICs and GMs take different physical mechanism although both of them have infinite Q-factors. BICs, also known as trapped modes, correspond to a special type of leaky modes with infinite life time and zero radiative decay rate despite coexisting into the radiation continuum^13^. Another intriguing property of BICs is that they can be linked with polarization singularities in the momentum space, which carry integer topological charges^14^. According to physical mechanism behind them, BICs can be divided into four categories: symmetry-protected BICs (SP-BICs)^15–19^, accidental BICs^20,21^, Friedrich-Wintgen BICs^22–26^, and Fabry-Perot BICs^13,27^. Among the diverse BICs, SP-BICs are particularly attractive because they can be easily accessed in a dielectric metasurface or photonic crystal slab whose lattices follow with certain group symmetry. SP-BICs usually happens at the Γ point of the first Brillouin zone^19^. Generally speaking, there are two ways of inducing the transition from BICs to quasi-BICs (QBICs). One is to apply oblique incidence^15,28,29^. Typically, the Q-factors of QBICs drop significantly with the wavevector in the momentum space. Another way is to introduce symmetry breaking in the periodic photonic structure^30–33^. When the structural symmetry of these photonic devices is disrupted, they are converted into QBICs with ultrahigh Q-factors^34–36^, which are also referred to as leaky modes. Different types of symmetry breaking within the unit cell, including notched nanodisks^37^, off-center-hole nanodisks^38^, T-shaped nanodisks^31^, and L-shaped nanodisks^39^, have been introduced to achieve high-Q QBICs. It has been well established that the Q-factors of QBICs are inversely proportional to the square of the asymmetry parameter^19^. However, realizing ultrahigh-Q QBICs is challenging because it is very difficult to obtain an ultra-small asymmetry parameter. In a word, the Q-factors of QBICs are extremely sensitive to the asymmetry parameters and incident angles.

Concurrently, another type of non-radiative modes present in metasurfaces is known as GMs, which are usually located below the light cone. GMs can be transformed into guided mode resonances (GMRs) with high Q-factors by incorporating periodic air holes into thin films^40,41^ or fabricating nanostructures array such as nanodisks^42^ and gratings^43^. Unlike BICs, GMs do not exhibit polarization singularities, and thus they do not carry integer topological charges. Furthermore, the Q-factors of GMRs remains robust in momentum space and shows less sensitivity to the incident angles. More recently, supercell composite metasurfaces have triggered extensive interest in nanophotonic community as they can simultaneously provide multiple BICs and GMs by leveraging Brillouin zone folding^44–50^. Remarkably, the super unit cell of such metasurfaces are usually made of dimer (2 × 1) or tetramer (2 × 2) nanodisks (or nanoholes). Compared to metasurfaces made of an array of single meta-atoms, composite metasurfaces provide much more freedom, including the lattice constants and geometry parameters (i.e., size, shape, and location) of nanodisks or nanoholes within the super unit cell, to tailor the high-Q resonances arising from either BICs or GMs. Given abundant parameters involved in composite metasurfaces, whether there are new types of BICs and GMRs with infinite Q-factors remain elusive. Furthermore, how to construct multiple ultrahigh-Q resonances in composite metasurfaces remains largely unexplored.

In this work, we present the theoretical prediction and experimental observation of two types of non-radiated modes in tetramer composite metasurfaces, one of which is nanoholes’ center point group SP-BICs and the other is diagonal holes’ area-preserved GMRs. We show that such tetramer composite metasurfaces support multiple SP-BICs at Γ points as long as the centers of four holes are located within the center of sub-unit cell regardless of the size and the shape of the air holes. When the center of a nanohole is displaced from its original position, these BICs are transformed into high-Q QBICs. Furthermore, we also demonstrate that multiple ultrahigh-Q GMRs (Q > 10^8^) can be achieved by maintaining area conservation among diagonal nanoholes within the supercell. Any area detuning could transform these modes into high-Q leaky modes. Notably, these modes typically exhibit degenerate characteristics and can yield polarization-dependent dual high-Q modes upon symmetry breaking within the supercell. Finally, we fabricated a series of silicon metasurfaces and employed a cross-polarization measurement system to experimentally validate both types of ultrahigh-Q resonances, where the maximum Q-factor is up to 43,702. Our work provides an alternative approach to obtaining ultrahigh-Q resonances in composite metasurfaces, and could be beneficial to developing high-performance photonic devices based on ultrahigh-Q resonators.

## Results

**Fig. 1:**
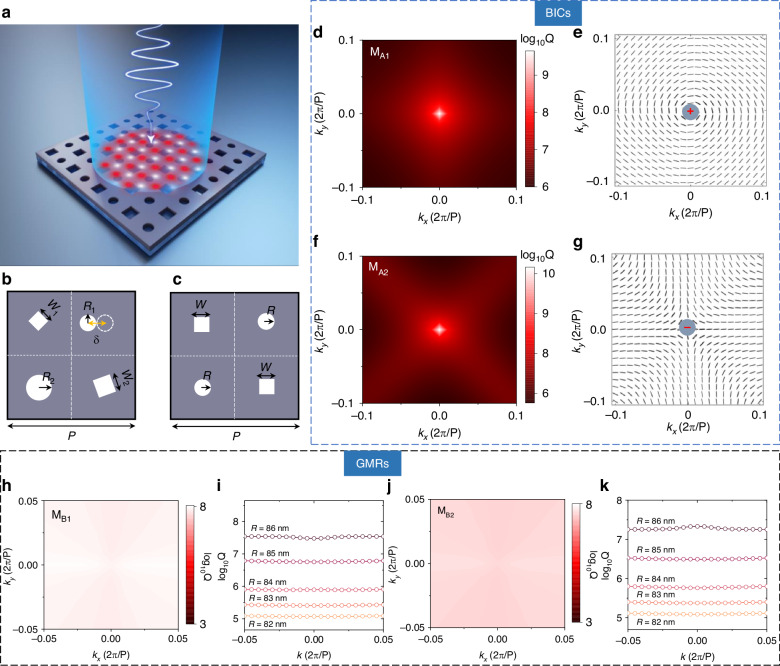
BICs and GMRs supported by the metasurface of composite supercells. **a** Schematic representation illustrating the light field trapping of nanohole tetramer structures. **b**, **c** Depictions of nanohole supercells, which support non-radiative BIC and GMR modes under conditions of centroid symmetry protection and diagonal area conservation. **d**, **f**, **h**, **j** Distribution of Q-factors in momentum space, revealing the modes associated with BICs and GMRs supported by composite nanohole structures. **e**, **g** Polarization vectors in *k*-space; **i**, **k** Variation of Q-factor as a function of *k* value for different hole radii

We start by investigating the eigenmodes in composite metasurfaces with a square lattice, which consist of an array of tetramer nanoholes penetrated through the silicon thin film. The refractive index of silicon is *n* = 3.48. These resonance modes are usually characterized by complex eigenfrequencies ω=ω_0_-iγ, where ω_0_ and γ are resonant frequency and radiative decay rate, respectively. Note that the complex eigenfrequencies and eigenfield distributions of eigenmodes are calculated with commercial software COMSOL Multiphysics based on finite element method (See Methods Section). Figure 1a presents a schematic diagram of the composite metasurface. When arranged in a specific configuration, the composite nanohole metasurface supports a series of non-radiative eigenmodes with infinite Q-factors, including BICs and GMs. Introducing a tiny perturbation can lead to the generation of high-Q leaky modes. To make our conclusion as general as possible, we focus on two types of super unit cell, as shown in Fig. 1b, c. We found that multiple BICs are supported when the centers of four nanoholes are located with the center of each sub unit cell in Fig. 1b. In other words, the nature of SP-BICs is preserved when the center-to-center distance between neighbor air holes is equal to the half of lattice constants of the composite metasurface. Nevertheless, exciting QBICs with high Q-factors occur when one nanohole is displaced from its original position. Considering a free standing silicon metasurface with tetramer air holes as an example, the relevant geometry parameters are period *P* = 800 nm, the side lengths of the two square hole *w*_1_ = 100 nm and *w*_2_ = 150 nm, with rotations set at angles of 45° and 20°, respectively. The radii for circular holes are *R*_1_ = 60 nm and *R*_2_ = 90 nm, while thickness is maintained at *h* = 220 nm. Here, it is worth noting that the size and shape of air holes are different to break the structural symmetry of the unit cell. Surprisingly, we can still find that such a tetramer metasurface accommodates two classes of BIC modes within the C-band range. Their Q-factors distributions calculated in momentum space are illustrated in Fig. 1d, f. These two modes are designated as M_A1_ and M_A2_ respectively—both exhibiting infinite Q-factors at Г point along with divergent distributions in *k*-space. To confirm that they are indeed BICs, we calculate polarization vectors mapping within first brillouin zone in Fig. 1e, g. Further calculation reveals that topological charges of two BICs are +1 for M_A1_ and -1 for M_A2_. More details of topological charge calculation are presented in Section I of Supplementary Materials. Notably, even when alterations occur to hole shapes, both BICs remain present—demonstrating their robustness against the shape of air hole. For a composite metausrface with super unit cell shown in Fig. 1c, we show that it supports some GMRs with ultrahigh Q-factors when the area sum of diagonal holes is equal to each other. The structural parameters are *w* = 150 nm, *R* = 86 nm, and *P* = 920 nm. Two typical examples of ultrahigh-Q GMRs labeled as M_B1_ and M_B2_, are presented in Fig. 1h, j. Different from BICs in Fig. 1d–g, these ultrahigh-Q modes do not carry topological charges as they cannot link with polarization singularities. Moreover, the Q-factors of these two modes are relatively stable within a wide range of wavevector, which is also another typical feature of GMRs. We also calculate the Q-factors of GMRs at different *R*, which are shown in Fig. 1i, k. Obviously, both modes show weak dependence on the wavevector. Also, when the radius *R* deviates from the critical radius, their Q-factors decrease.

**Fig. 2:**
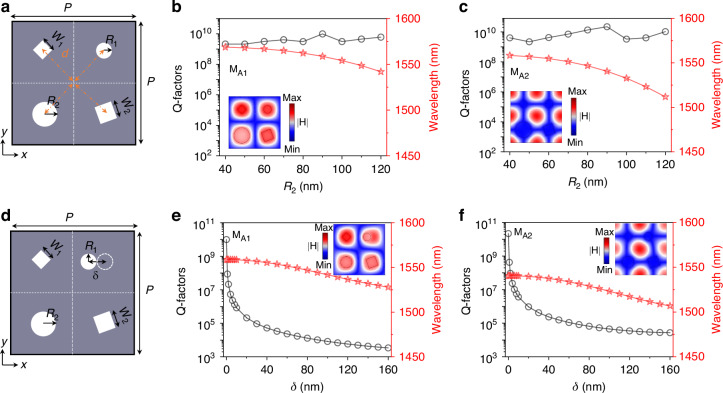
Centroid symmetry breaking excitation of QBIC modes. **a** Schematic representations of tetramer supercells featuring various nanohole geometries. **b**, **c** The Q-factors and wavelengths of the two BIC modes as a function of increasing radius *R*_2_ of the circular hole, accompanied by corresponding eigenfield illustrations. **d** A schematic diagram illustrating the supercell configuration when one of the circular holes experiences a relative positional shift. **e**, **f** Variation in Q-factors and wavelengths for the two QBIC modes with respect to offset δ, the inset displays the magnetic field distribution associated with excited quasi-BICs

Next, we explore the excitation mechanisms and properties of these two types of modes in details. We first consider the case of SP-BICs in tetramer composite metasurfaces, as schematically shown in Fig. 2a. The structural parameters are the same as those in Fig. 1b. As previously mentioned, BICs are independent of the geometrical configuration of the four nanoholes. This is because the center point group symmetry of the sub unit cell still follows C_4v_ although the structural symmetry is broken. Strictly speaking, as long as four air holes are located in the center of sub unit-cell, the eigenfields’ symmetry for both modes are maintained (See Figure S1), signifying the preserved nature of BICs. To further confirm that the nature of SP-BICs is indeed preserved, we calculate the Q-factors of mode M_A1_ and M_A2_ by varying *R*_2_ while the other parameters are fixed. It is observed that the Q-factor remains nearly constant and exceeds 10^9^, thereby consistently preserving the robustness of BICs. Meanwhile, as the hole radius *R*_2_ increases, both resonance wavelengths experience blueshift because of the reduced dielectric materials, as depicted in Fig. 2b, c. To explore deeper insight of physical nature of these two modes, we calculate eigenfield distributions of two modes in Figures S2a, b. One can see that the eigenfield profiles remain almost the same, confirming the preserved nature of BICs. Except for the stably high Q-factors, we also find that the eigenfield profiles of two modes exhibit drastically different behavior although both modes are TE-like modes. For M_A1_, the maximum of magnetic fields are located within and around four air holes. However, they are distributed within the silicon film for mode M_A2_. This distinction suggests different potential application scenarios for each mode. If one of the nanoholes is displaced—thereby disrupting C_4v_ symmetry—as shown in Fig. 2d–f, both modes experience a sharp decline in their Q-factors with the increasing offset. This signifies a transition from BIC modes to QBIC leaky modes. This trend can be well explained from eigenfield perturbation perspective (See bottom panels of Figures S2a, b, where eigenfield distributions is severely perturbed by the displacement of air hole). These QBIC modes are characterized by finite yet high Q-factors that can be directly coupled with incident light to enhance light-matter interactions effectively. Besides, it can be found that the Q-factors of mode M_A2_ is slightly higher than M_A1_ for the same offset distance. This is because the eigenfield profiles are less perturbed for M_A2_ compared to the counterpart of M_A1_ when the air hole moves along *x*-axis. Besides, we perform multipole decomposition to reveal the nature of these two modes^51,52^ (See Fig. S3). Multipolar analysis show that both modes are dominated by magnetic dipole (MD), followed by electric quadrupole (EQ). Additionally, such BICs can also be found in silicon metasurfaces whose unit cell is made of four nanoparticles (See Figs. S4, 5).

**Fig. 3:**
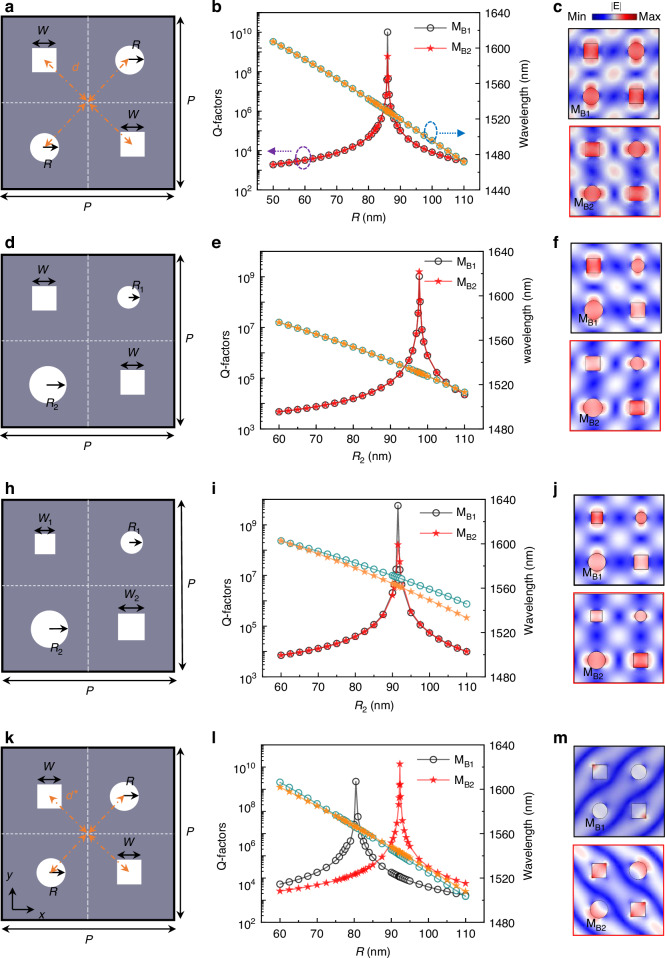
High-Q GMR properties related to diagonal nanoholes area conservation in supercells. **a**, **d**, **h**, **k** illustrate four distinct structural configurations for supercells: **a** features a diagonal circular hole area that matches in area with a square hole, **d** maintains equal sizes for both square holes while varying those for circular holes, **h** presents inconsistencies among sizes across four nanoholes devices, **k** shows four holes converging towards or diverging from the center of the supercell. **b**, **e**, **i**, **l** depict variations in Q-factor and wavelength associated with parameters *R* and *R*_2_. Herein, d = $\frac{\sqrt{2}{\rm{P}}}{4}$ in (**b**, **e**, **i**), d*=d-10$\sqrt{2}$ in (**l**). **c**, **f**, **j**, **m** display eigenfield distributions corresponding to the two ultrahigh-Q GMRs

Then, we move to discuss the excitation of ultrahigh-Q GMRs, as illustrated in Fig. 3a. The supercell comprises two circular and square holes (*w* = 150 nm) and maintains a symmetric distribution characterized by d_0_ = $\frac{\sqrt{2}{\rm{P}}}{4}$, thereby satisfying C_2_ symmetry. The lattice constants are *P* = 920 nm. Note that four holes are located at the center of sub unit cell. If we set the center of super unit cell as origin, the coordinates of four holes’ center are (±*P*/4, ±*P*/4). Such a metasurface could support multiple ultrahigh-Q GMRs while preserving the conservation of the diagonal nanohole area in the supercell. As shown in Fig. 3b, two high-Q GMRs (M_B1_ and M_B2_) are degenerate, and their Q-factors approach infinity when *R* increases to *R*_0_ = 86 nm. Carefully checking the diagonal holes area suggests that the area sum of two circular holes is almost the same as those of square hole (${\rm{\pi }}{R}_{0}^{2}\approx {w}^{2}$). That is why we call these two ultrahigh-Q GMRs as area-conservation preserved high-Q modes. By deviating *R* from *R*_0_, we can convert these modes with almost infinite Q-factors into leaky modes with finite but high Q-factors. One can also find that eigenfield profiles are drastically different from the case of GMRs with almost infinite Q-factors, where electric fields tend to localize within the circular air hole (See Fig. S2c, d). Multipole decomposition indicates that these two modes are dictated by toroidal dipole (TD), magnetic quadrupole (MQ) also makes considerable contributions (See Fig. S6a–d). From Fig. 3c, it can be observed that the eigenfields of both modes reside in air regions and rotate by 90°, confirming their degeneracy. Furthermore, we shall point out that such area-conserved ultrahigh-Q GMRs still exist even when two circular air holes have different radius. As shown in Fig. 3d–f, we demonstrate that upon reducing one circular hole’s size (*R*_1_ = 70 nm) while adjusting only the other circular hole’s size (*R*_2_), two ultrahigh-Q GMRs still emerge at *R*_2_ = 97.8 nm, where area conservation (${\rm{\pi }}{R}_{2}^{2}\approx 2{w}^{2}-{{\pi }R}_{1}^{2}$) is maintained. Furthermore, their degenerate feature is preserved. This can be well explained by choosing appropriate super unit cell of composite metasurface (See Figure S7). It is interesting to note that such degenerate ultrahigh-Q GMRs can be lifted by further breaking the structural symmetry. Via adjusting width differences in square holes (*w*_1_ = 150 nm, *w*_2_ = 120 nm) while *R*_1_ is fixed at *R*_1_ = 60 nm. From Fig. 3h–j, it can be observed that the ultrahigh-Q GMRs still appears at the same *R*_2_ = 91.5 nm, where the area sum of two diagonal holes are conserved (${{\pi }R}_{1}^{2}+{\rm{\pi }}{R}_{2}^{2}\approx {w}_{1}^{2}+{w}_{2}^{2}$). A notable distinction is that as *R*_2_ increases, the resonance wavelengths of the two modes gradually split, corresponding to non-degenerate dual-wavelength resonances. Multipole decomposition results suggest that mode M_B1_ is still governed by TD and MQ. For mode M_B2_, TD makes greatest contribution, followed by MD, MQ and EQ (See Figure S6e–h). Here, it is worth noting that such area-conservation induced ultrahigh-Q GMRs can be found even when *d* deviates from d_0_ = $\frac{\sqrt{2}{\rm{P}}}{4}$. In other words, four holes are no longer located at the center of four sub unit cell. Their centers move either toward or outward the origin of the super unit cell. Under such circumstances, the critical radius *R*_2_ for the two ultrahigh-Q GMRs are no longer the same while the resonance wavelengths of two modes are different, as shown in Fig. 3k–m. Additionally, we shall point out that such a metasurface also support the other degenerate ultrahigh-Q modes characterized by area conservation in the O-band, where the field distributions are primarily localized within the materials (See Figure S8).

**Fig. 4:**
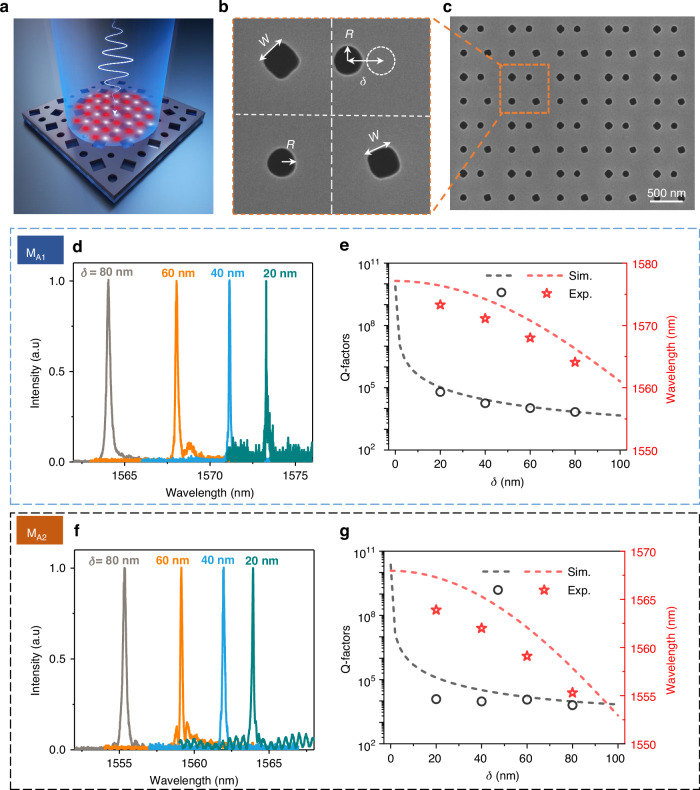
Experimental validation of high Q-factor QBICs in supercell composite metasurfaces with centroid symmetry breaking. **a** Schematic illustration of QBICs excitation in the supercell device. **b**, **c** SEM images of the typical samples, the position offset of one of the circular holes is denoted by δ. **d**, **f** Normalized reflection intensity spectra for modes M_A1_ and M_A2_, measured experimentally at different δ values. **e**, **g** Comparative plots of Q-factors and wavelengths for the two modes, obtained from both experimental data and simulations at varying δ values

After gaining solid understanding of ultrahigh-Q modes in tetramer composite metasurfaces, we move to demonstrating such two types of high-Q resonances experimentally by fabricating a series of metasurfaces based on silicon-on-insulator (SOI) wafer and characterizing them with cross-polarization measurement system. The metasurfaces are fabricated with standard nanofabrication technology (See Methods Section), including electron beam lithography and inductively coupled plasma etching. Here, we first consider excitation of QBICs induced by center displacement of one air hole, as schematically shown in Fig. 4a. The structure parameters of fabricated devices are *P* = 800 nm, *w* = 125 nm, *R*_1_ = *R*_2_ = *R* = 60 nm, and the two square holes are rotated clockwise by 20° and 45°, respectively. Figure 4b, c present a scanning electron microscope (SEM) image of one represented sample. Center point group symmetry is broken by introducing center offset into the supercell structure, as displayed in Fig. 4b. The offset δ are chosen as 20 nm, 40 nm, 60 nm, and 80 nm, respectively. Unlike the radius of air hole that can be varied by different etching recipe, in real fabrication such an offset can be accurately controlled, thus providing an easy way of tailoring the Q-factors of QBICs. Figure 4d, f show the normalized reflection intensity spectra for modes M_A1_ and M_A2_ at different offsets. It is observed that the linewidths of both modes gradually become narrower as δ increases, indicating a continuously increase in their Q-factor. At the same time, the resonance wavelengths show redshift with the increasing offset δ, possibly arising from the coupling between neighbor air holes within the super unit cell. We also retrieve the resonance wavelengths and Q-factors shown in Fig. 4e, g. Fano fitting can be found in Result and Fig. S9 of Supplementary Materials. Excellent agreement can be found between experiments and simulations for both resonance wavelengths and Q-factors. Significantly, while all of measured Q-factors are above 10,000, the highest experimental Q-factor is up to 43,702 at δ = 20 nm (See Figure S9). One can obtain even higher Q-factors of QBICs at an even smaller δ. It is noteworthy that these metasurfaces also support other SP-BIC modes at shorter wavelength. Introducing symmetry breaking via moving one of the air holes also induces the transition from BICs to QBICs with high Q-factors. The experimental results are shown in Figure S10.

**Fig. 5:**
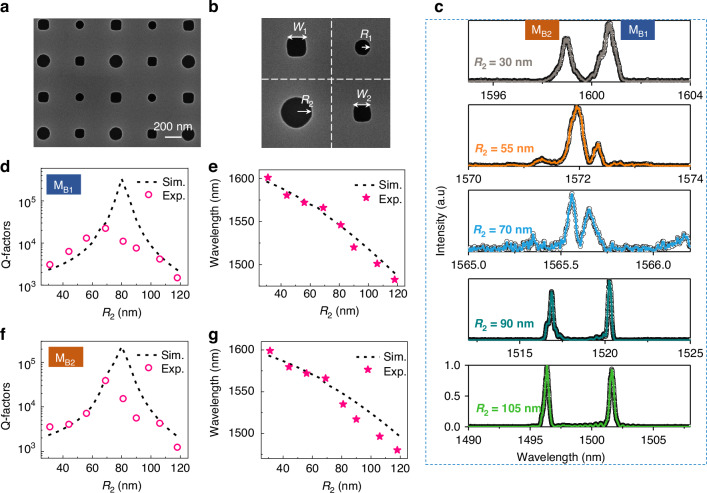
Experimental confirmation of high Q-factor GMRs induced by supercell diagonal area conservation perturbation. **a** SEM image of a typical sample. **b** SEM image and parameter notation for the super unit cell. **c** Normalized reflection intensity spectra of the sample as *R*_2_ is varied, showing the separation of two degenerate modes labeled M_B1_ and M_B2_. **d**–**g** Comparative analysis of experimental and simulated Q-factors and wavelengths for the two modes

Finally, we present an experimental confirmation of high-Q GMRs by introducing area conservation detuning of diagonal nanoholes in the supercell. Figure 5a shows the SEM image of one typical sample, while Fig. 5b illustrates the zoomed in SEM image of a supercell where the two square holes have different sizes. The structural parameters of the fabricated sample are *P* = 880 nm, *w*_1_ = 130 nm, *w*_2_ = 110 nm, and *R*_1_ = 56 nm. Only *R*_2_ is varied to observe the area-conservation induced ultrahigh-Q GMRs. We demonstrated that breaking the symmetry of the device lifts the degeneracy of modes M_B1_ and M_B2_. Figure 5c shows the reflection spectra at different *R*_2_, where two distinct resonant peaks are clearly observed due to nondegenerate resonant modes. Similarly, we also compare the Q-factors and wavelengths of the two non-degenerate modes in Fig. 5d–g. The experimental results show reasonably good agreement with simulations. However, due to deviations in nanohole size, there is a slight discrepancy between the experimental and designed devices in terms of diagonal area conservation. As a result, the critical radius *R*_2_ corresponding to the highest Q-factor in the experiment is slightly lower than the design value, and the maximum Q-factor achieved for GMRs is 15,350 (See Figure S9). The Q-factors’ difference between experiments and simulations is primarily attributed to imperfections in sample preparation, such as roughness, uniformity and sidewall verticality of nanoholes. Optimizing the sample preparation process will further enhance the Q-factor. It is noted that both cases-silicon metasurfaces on top of SiO_2_ substrate with infinite thickness and SOI platform-exhibit resonances with almost identical resonant wavelength and linewidth (see Figure S11).

## Discussion

In this work, we demonstrate that two types of ultrahigh-Q resonances, such as center point group SP-BICs and area-conservation preserved GMRs, can be simultaneously supported in tetramer composite metasurfaces. It is found that multiple SP-BICs are always preserved as long as the centers of four air nanoholes are located at the center of each sub unit cells regardless of their shapes and sizes. Topological charges and polarization vector mapping were calculated to verify the nature of these BIC. Upon deviation of one nanohole from its original position, the Q-factors of these modes rapidly decrease, indicating that BICs are successfully transformed into high-Q QBIC. Except for point group SP-BICs, we find that when such composite metasurfaces also host many GMRs with almost infinite Q-factors when the area sum of the diagonal nanoholes in the supercell is conserved. Unlike the case of BICs, such GMRs does not carry topological charge, and their Q-factors are relatively stable in momentum space. Introducing an area conservation detuning by altering the size of one nanoholes can effectively tailor the Q-factor of these modes, leading to the excitation of GMR with finite but high Q-factors. Notably, under C_4v_ symmetry conditions, these modes typically exhibit double degeneracy. Introducing asymmetry results in the separation of their eigenfrequencies, thus removing degeneracy. The QBICs and GMRs with high Q-factors were experimentally confirmed by fabricating a series of silicon metasurfaces and measuring their scattered spectra. Good agreement can be found between experimental measurements and numerical simulations. The highest experimental Q-factor is up to 43,702. Our findings offer a novel approach for exciting high-Q resonances in composite devices, thereby advancing the development of high-performance nanophotonic devices.

## Methods

### Numerical calculations

The Q-factors, complex eigenfrequencies, polarization vector, and eigenfields are calculated using the commercial software COMSOL Multiphysics, which employs the finite element method. The refractive indices for silicon and silicon dioxide are 3.48 and 1.45, respectively. Floquet periodic boundary conditions are implemented in both the *x*- and *y*-directions, while perfectly matched layers (PML) are utilized in the *z*-direction.

### Sample fabrication

The silicon metasurfaces are fabricated using an SOI wafer with a 220-nm-thick top silicon layer. Initially, a resist layer (ZEP520A) is spin-coated onto the clean SOI wafer. The patterns are then defined in resist layer through EBL. Subsequently, ICP etching is employed to transfer the metasurface pattern onto the top silicon layer. Finally, the remaining resist is removed using the methyl-2-pyrrolidone (NMP) solution. It should be noted that the metasurfaces are fabricated at Tianjin H-Chip Technology Group Corporation.

### Optical characterization

The scattering intensity spectra were measured using a home-built microscope spectroscopy system based on cross-polarization measurement (See Fig. S12). The tunable telecommunications laser (Santec TSL-550, 1480 nm–1630 nm) first passes through a polarizer, lens, and beam splitter before being focused onto the rear focal plane of the objective lens (10×), ensuring that the incident light on the sample is nearly normal incidence. The reflected signal are collected by a photodiode (PDA10DT-EC), which can be switched to a CCD for sample localization. A lock-in amplifier, in conjunction with a chopper (SR540), is connected to the photodiode to extract the signal from background noise. A pair of strictly orthogonal polarizers are positioned at both the entrance and exit of the light path to facilitate cross-polarization.

## Supplementary information

Robust Ultrahigh-Q Resonances in Tetramer Metasurfaces through Centroid Symmetry Protection and Area Conservation

## Data Availability

The main data supporting the findings of this study are available within the article and its Supplementary Information files. Extra data are available from the corresponding author upon reasonable request.
