# Supplementary material for: Robust ultrahigh-Q resonances in tetramer metasurfaces through centroid symmetry protection and area conservation
Source: Light Sci Appl. 2026 Jan 23;15:84. doi: 10.1038/s41377-025-02164-7 (PMC12830976; doi:10.1038/s41377-025-02164-7)
Supplement: Supplementary file 1 — Robust Ultrahigh-Q Resonances in Tetramer Metasurfaces through Centroid Symmetry Protection and Area Conservation [file 41377_2025_2164_MOESM1_ESM.pdf]

Supplementary Information for

Robust Ultrahigh-Q Resonances in Tetramer Metasurfaces through Centroid Symmetry Protection and Area Conservation

Chaobiao Zhou[1,#,*], Rong Jin[2,3,4,#], Haoxuan He[1], Jing Huang[1], Guanhai Li[2,3,4,*], Lujun Huang[5, *]

[1]*School of Physics and Mechatronic Engineering, Guizhou Minzu University, Guiyang, 550025, China*

[2]*State Key Laboratory of Infrared Physics, Shanghai Institute of Technical Physics, Chinese Academy of Sciences, 500 Yu Tian Road, Shanghai 200083, China.*

[3]*Hangzhou Institute for Advanced Study, University of Chinese Academy of Sciences, No.1 SubLane Xiangshan, Hangzhou 310024, China.*

[4]*Shanghai Research Center for Quantum Sciences, 99 Xiupu Road, Shanghai 201315, China*

[5]*State Key Laboratory of Precision Spectroscopy, School of Physics and Electronic Sciences, East China Normal University, Shanghai 200241, China*

#C. Zhou and R. Jin contributed equally to this work.

*Corresponding author: *Email: cbzhou@gzmu.edu.cn; ghli0120@mail.sitp.ac.cn; ljhuang@phy.ecnu.edu.cn*

Section I: Calculation of topological charge

Section II: Fano resonance fitting

Figures S1-S12

Section I: Calculation of topological charge

The topological charge associated with this vortex is defined as the winding number of the polarization vector, which can be expressed as [1,2]:

$$q = \frac{1}{2\pi}\oint_C d\mathbf{k}_{||} \cdot \nabla_{\mathrm{k}} \phi(\mathbf{k}_{||}),$$

where C denotes the loop integral around the center of the vortex in a counterclockwise direction. Here, $\phi(\mathbf{k}_{||}) = \arg\left[c_x(k_{||}) + ic_y(k_{||})\right]$ represents the angle between the wave vector and its projection onto the plane-defined polarization vector, while $c_x(k_{||})$ and $c_y(k_{||})$ denote its components along the x and y axes, respectively.

Section II: Fano resonance fitting

The Fano resonance can be fitted by the classical Fano formula [3,4]:

$$T(\omega) = T_0 + A_0 \frac{[q + 2(\omega - \omega_0)/\Gamma]^2}{1 + [2(\omega - \omega_0)/\Gamma]^2},$$

where ω_0 is the resonant frequency, Γ is the resonance linewidth, and T_0 is the background scattering parameter, A_0 is the continuum-discrete coupling constant, q is the Breit-Wigner-Fano parameter determining asymmetry of the resonance profile. The Q-factor is evaluated by $\frac{\omega_0}{\Gamma}$.

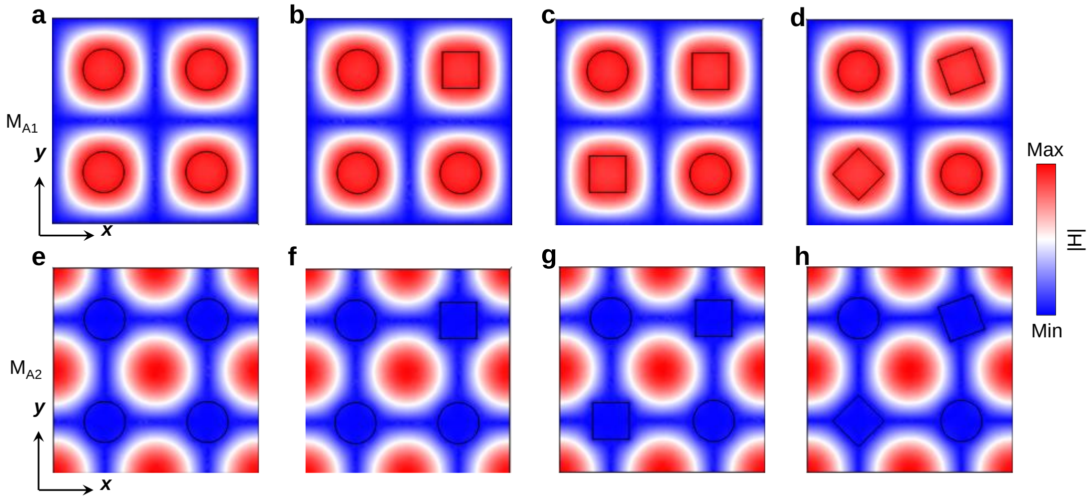

Figure S1. (a-d) Eigenfield distributions of mode M_{A1} for super unit cell made of four identical circular holes (a), three circular holes and one square hole (b), two circular and two square holes (c), two circular holes and two rotated square holes (d). (e-h) Eigenfield distributions of mode M_{A2} for super unit cell made of four identical circular holes (e), three circular holes and one square hole (f), two circular and two square holes (g), two circular holes and two rotated square holes (h).

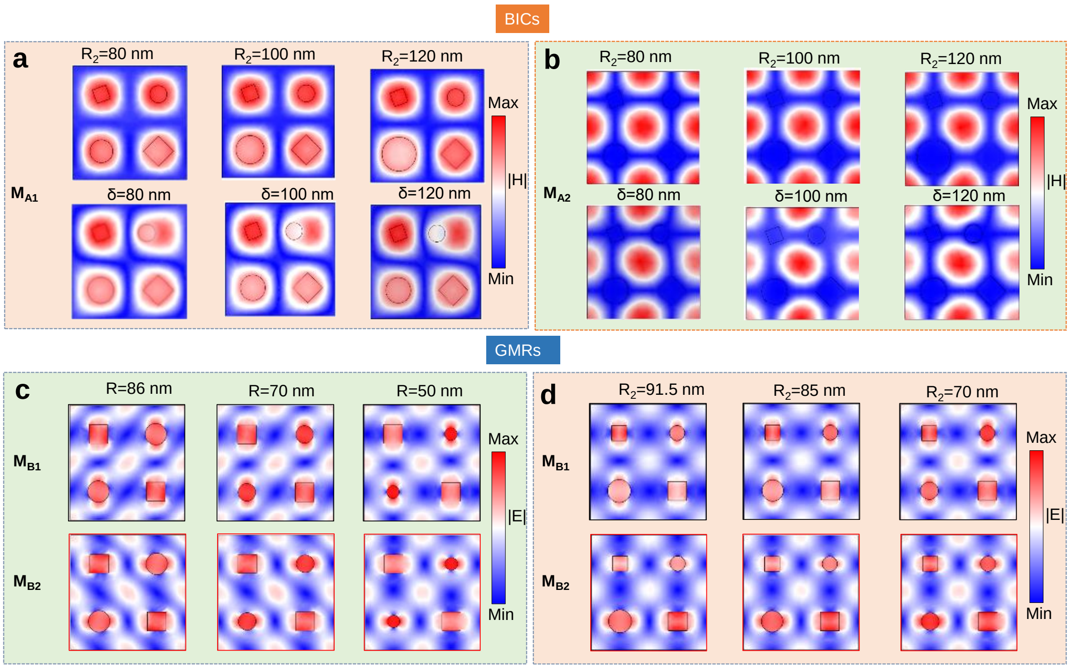

Figure S2. (a-b) Eigenfield profiels of modes M_{A1} and M_{A2} for metasurfaces with different R_2 or δ . (c-d) Eigenfield profiels of modes M_{B1} and M_{B2} for metasurfaces with two identical square holes (c) and two different square holes (d) at different R and R_2.

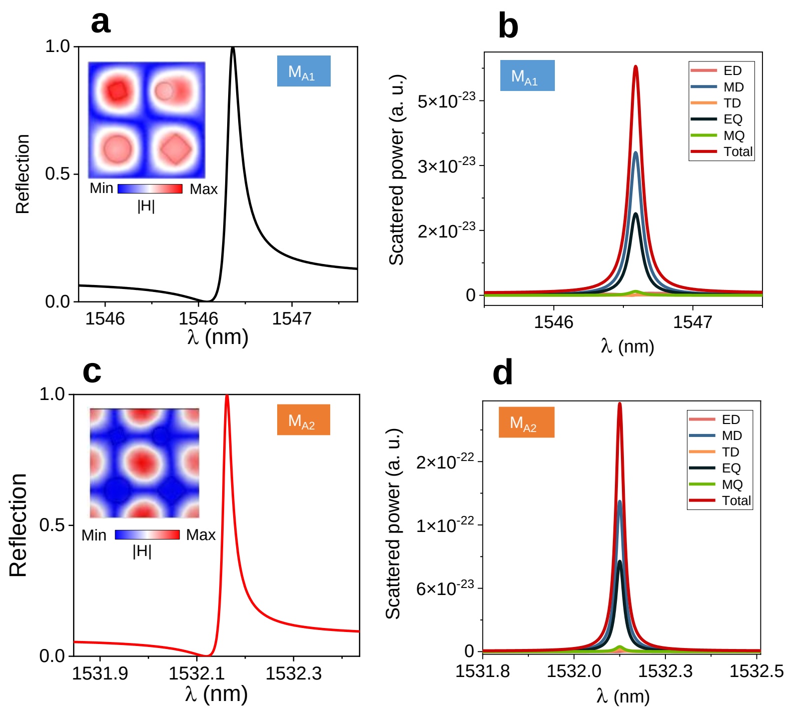

Figure S3. (a-b) Reflection spectra (a) and multipolar decomposition results (b) for M_{A1} modes under δ= 80 nm. (c-d) Reflection spectra (c) and multipolar decomposition results (d) for M_{A2} modes under δ= 80 nm.

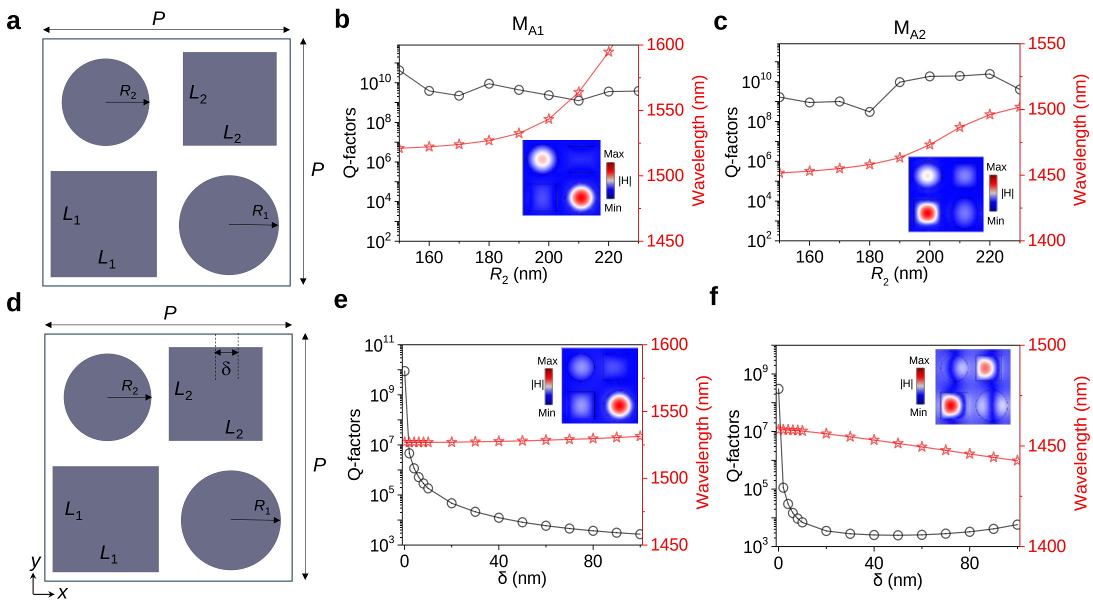

Figure S4. (a) Schematic drawing of the unit cell of silicon metasurfaces with P=1000 nm, L_1=360 nm, L_2=340 nm and R_1=210 nm. (b) The Q-factors and wavelengths of Mode M_{A1} vs R_2. (c) The Q-factors and wavelengths of mode M_{A2} vs R_2. (d) Schematic drawing of the unit cell of silicon metasurfaces with P=1000 nm, L_1=360 nm, L_2=340 nm, R_1=210 nm, and R_2=180 nm. (e) The Q-factors and wavelengths of mode M_{A1} vs δ. (f) The Q-factors and wavelengths of mode M_{A2} vs δ.

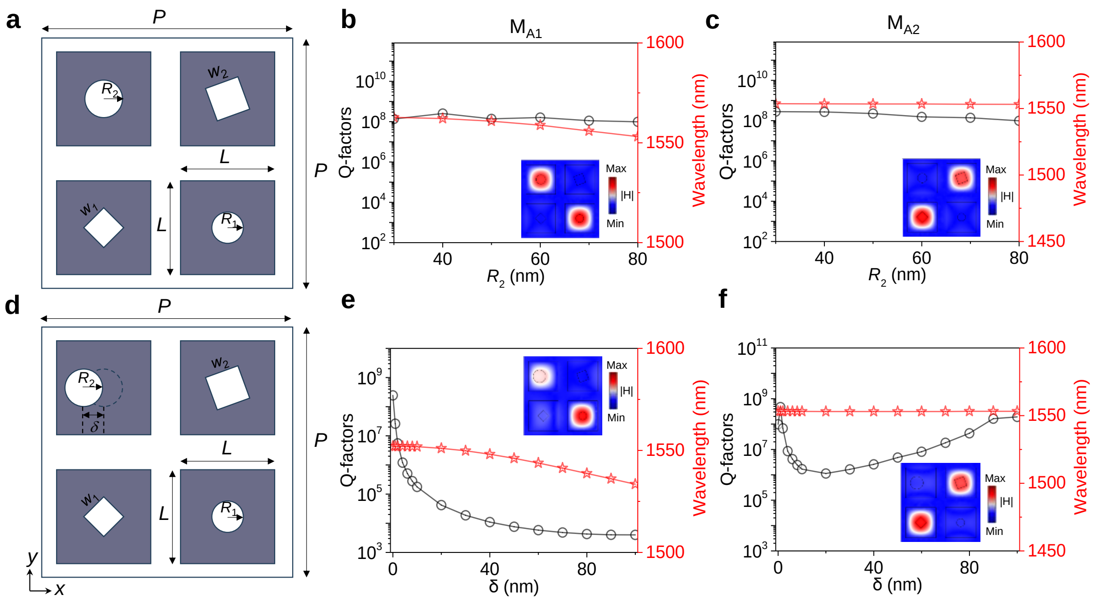

Figure S5. (a) Schematic drawing of the unit cell of silicon metasurfaces with P=1000 nm, L=380 nm, w_1=100 nm, w_2=120 nm and R_1=50 nm. (b) The Q-factors and wavelengths of mode M_{A1} vs R_2. (c) The Q-factors and wavelengths of Mode M_{A2} vs R_2. (a) Schematic drawing of the unit cell of silicon metasurfaces with P=1000 nm, L=380 nm, w_1=100 nm, w_2=120 nm, R_1=50 nm, and R_2=50 nm. (b) The Q-factors and wavelengths of mode M_{A1} vs δ. (c) The Q-factors and wavelengths of mode M_{A2} vs δ.

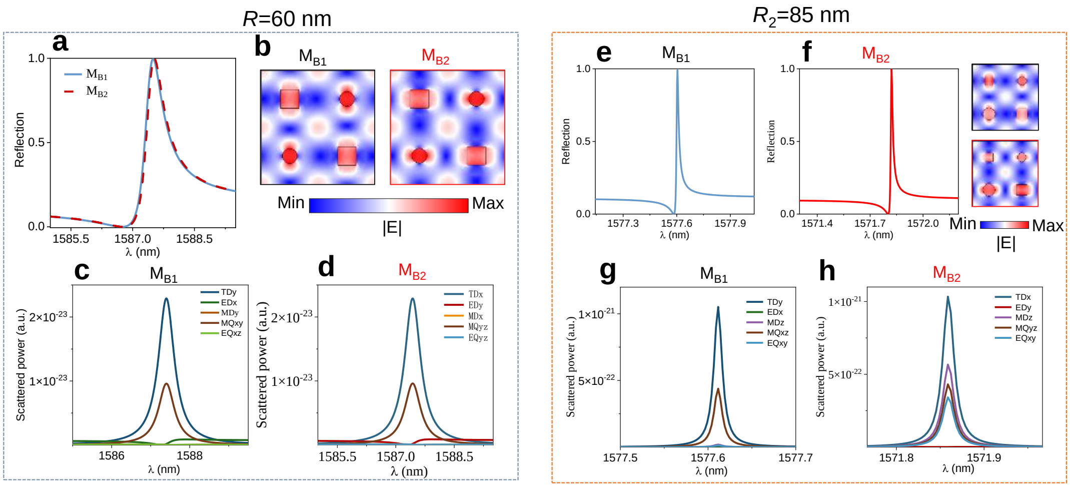

Figure S6. (a-d) Reflection spectra (a), eigenfield distributions of mode M_{B1} and M_{B2} (b), multipolar decomposition results of mode M_{B1} (c) and M_{B2} (d) for silicon metasurfaces with R=60 nm and w=150 nm. (e-h) Reflection spectra of M_{B1} (e) and M_{B2} (f), multipolar decomposition results of mode M_{B1} (g) and M_{B2} (h) for silicon metasurfaces with R_1=60 nm, w_1=150 nm, and w_2=120 nm, the eigenfield distributions of mode M_{B1} and M_{B2} are shown in right panel of (f).

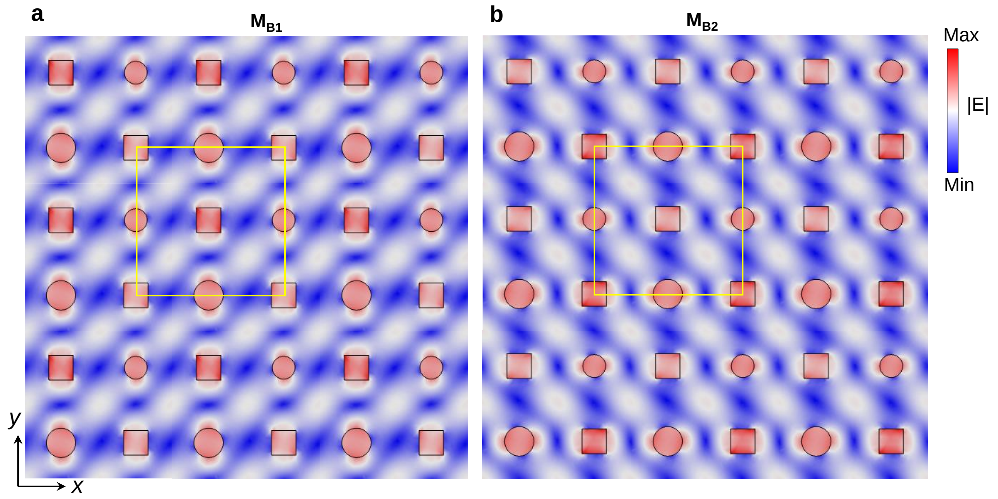

Figure S7. (a-b) Eigenfield profiles of modes M_{B1} (a) and M_{B2} (b) in 3×3 super unit cells.

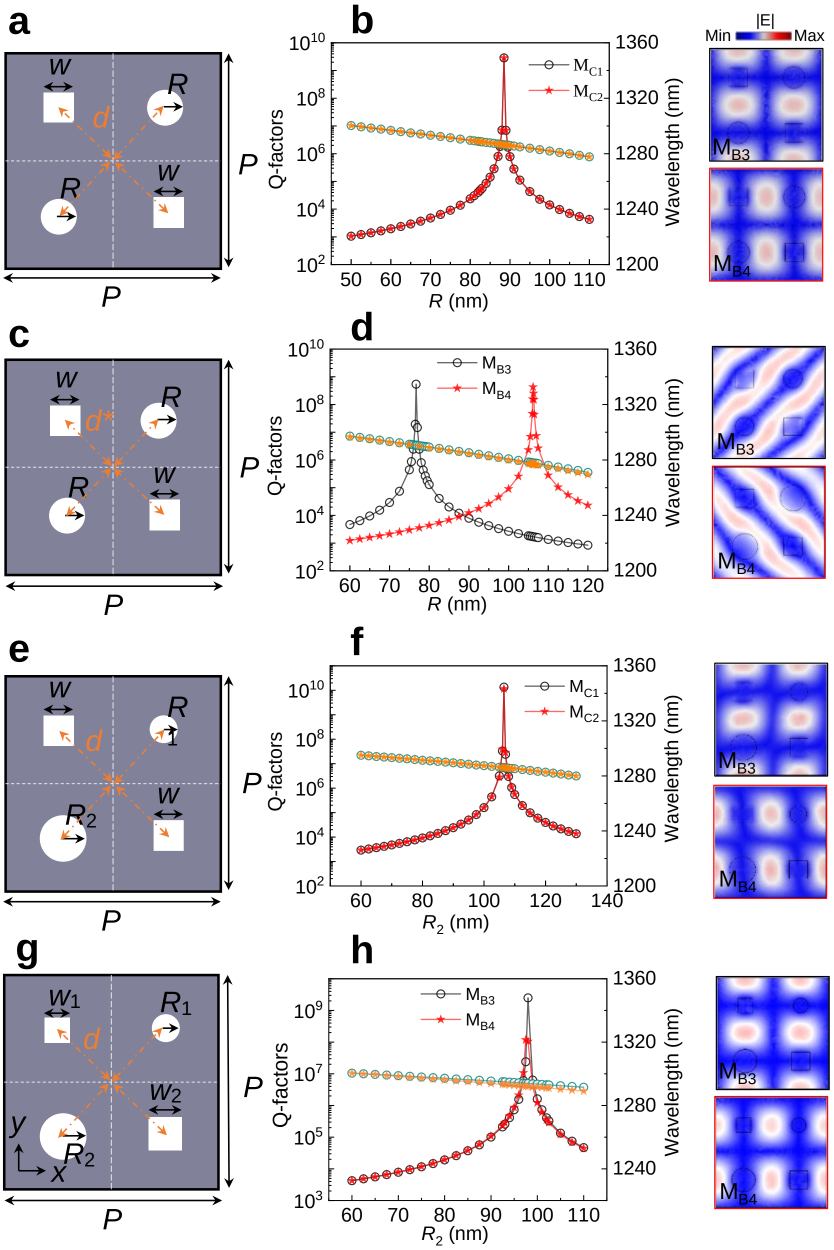

Figure S8. Characteristics of two ultra-high Q GMRs in the O-Band. (a), (c), (e), and (g) illustrate four distinct supercell structures. Specifically, (a) depicts a configuration where the diagonal circular hole and square hole possess identical sizes, accompanied by symmetrically distributed nanopores ($d = \frac{\sqrt{2}P}{4}$). In (c), the holes are situated closer to the center, with $d^* = d - 10\sqrt{2}$. (e) presents structures featuring uniform square holes while exhibiting variability in the size of circular holes. Finally, (g) show a structure wherein all four nanohole devices differ in size. (b), (d), (f), and (h) display the Q-factors and wavelengths corresponding to variations in R and R_2, along with the eigenfield distribution of two modes at ultra-high GMRs. Electric field of the two modes are predominantly localized within the device, distinguishing it from other GMRs discussed in main text. The observed characteristics of Q-factor and corresponding wavelength are consistent with the GMRs presented in text.

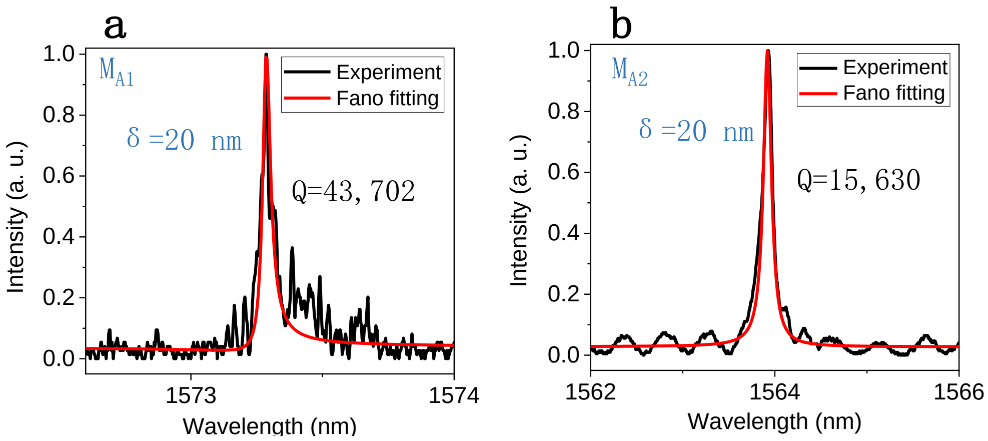

Figure S9. Reflection intensity spectra for high Q resonances fitted using the Fano formula, with the highest Q-factor reaching 43,702.

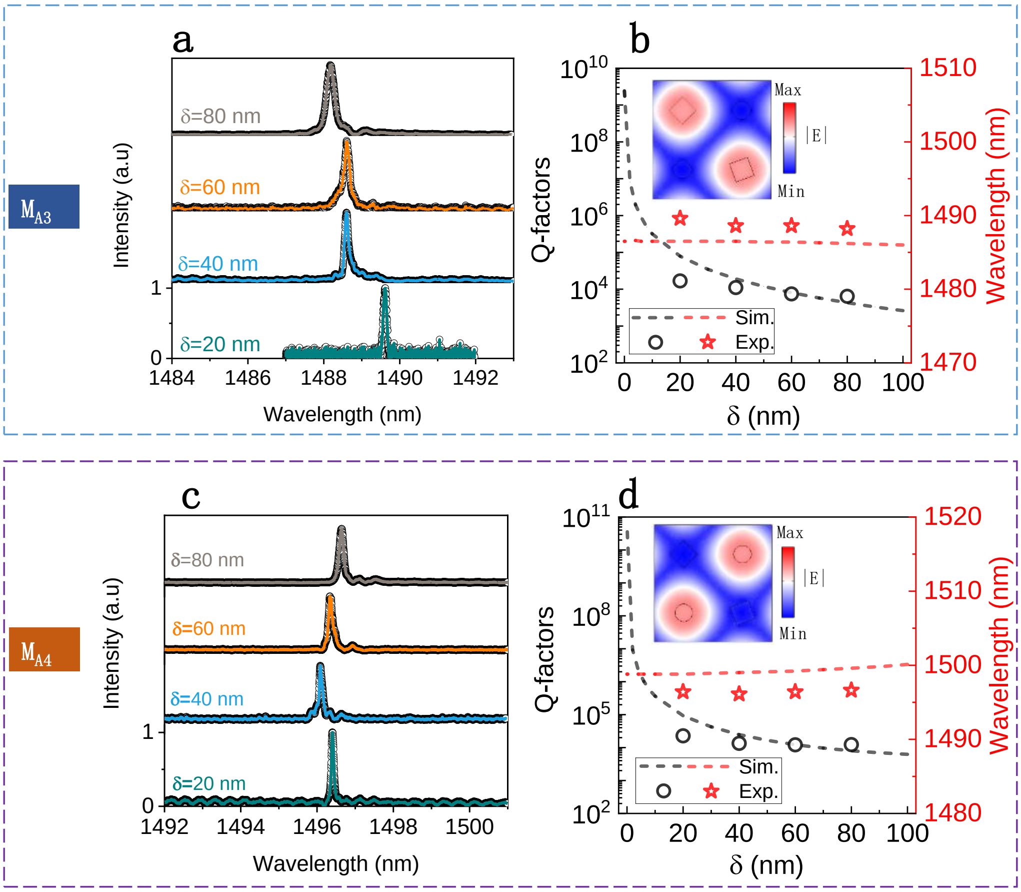

Figure S10. Experimental confirmation of the other two BICs. (a), (c) Normalized reflection intensity spectra for modes M_{A3} and M_{A4}, measured experimentally at different δ values. (b, (d) Comparative plots of experimental and simulated Q-factors and wavelengths for the two modes for varying δ values.

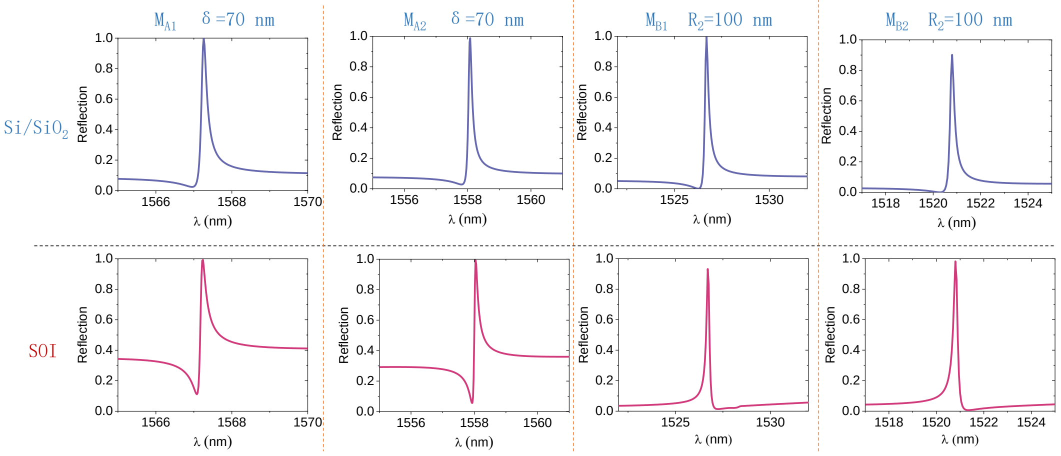

Figure S11. Reflection spectra of silicon metasurfaces on top of SiO_2 substrate and 2 μm SiO_2/Si, respectively.

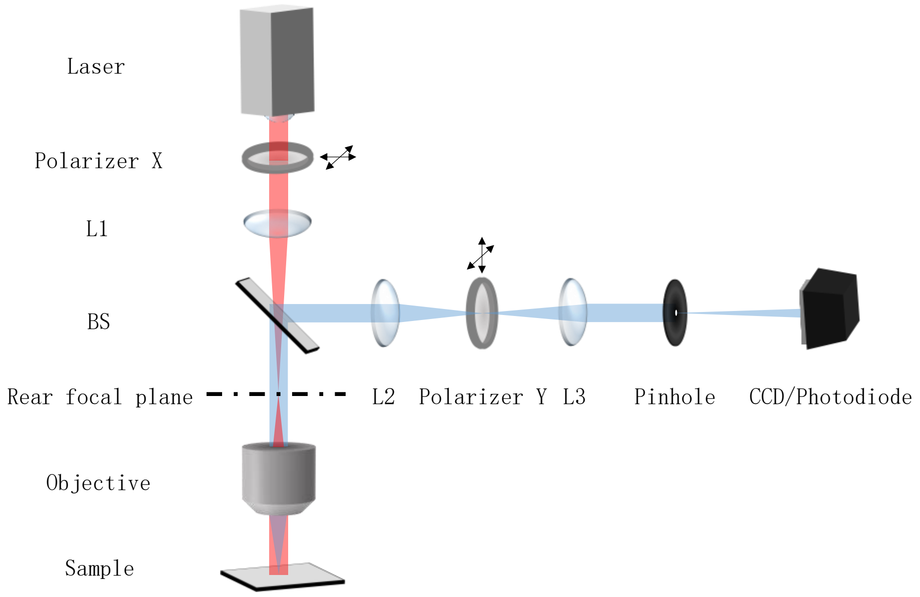

Figure S12. Schematic illustration of the experimental setup. The red and blue lines represent the incident light and the scattered light from the sample, respectively. “L”s denote lenses, while “BS” refers to beam splitter.

References

[1] B. Zhen, C. W. Hsu, L. Lu, *et al.* Topological nature of optical bound states in the continuum. Phys. Rev. Lett., 2014, 113: 257401.

[2] K. Koshelev, S. Lepeshov, M. Liu*, et al.* Asymmetric metasurfaces with high-Q resonances governed by bound states in the continuum. Phys. Rev. Lett., 2018, 121: 193903.

[3] W.X. Lim, M. Manjappa, P. Pitchappa and R. Singh, Shaping high-Q planar Fano resonant metamaterials toward futuristic technologies. *Adv. Opt. Mater.*, 2018. *6*: 1800502.

[4] A.E. Miroshnichenko, S. Flach, Y.S. Kivshar. Fano resonances in nanoscale structures. *Rev. Mod. Phys.*, 2010, 82: 2257-2298.
